# Simulating Arbitrary Electrode Reversals in Standard 12-Lead ECG

**DOI:** 10.3390/s19132920

**Published:** 2019-07-01

**Authors:** Vessela Krasteva, Irena Jekova, Ramun Schmid

**Affiliations:** 1 Institute of Biophysics and Biomedical Engineering, Bulgarian Academy of Sciences, Acad. G. Bonchev Str. Bl 105, 1113 Sofia, Bulgaria; irena@biomed.bas.bg; 2 Signal Processing, Schiller AG, Altgasse 68, CH-6341 Baar, Switzerland; ramun.schmid@schiller.ch

**Keywords:** ECG electrode swaps, ECG electrode potentials, WCT potential change, reconstructing correct ECG leads, MSMinv transformation, unicolor limb–chest electrodes

## Abstract

Electrode reversal errors in standard 12-lead electrocardiograms (ECG) can produce significant ECG changes and, in turn, misleading diagnoses. Their detection is important but mostly limited to the design of criteria using ECG databases with simulated reversals, without Wilson’s central terminal (WCT) potential change. This is, to the best of our knowledge, the first study that presents an algebraic transformation for simulation of all possible ECG cable reversals, including those with displaced WCT, where most of the leads appear with distorted morphology. The simulation model of ECG electrode swaps and the resultant WCT potential change is derived in the standard 12-lead ECG setup. The transformation formulas are theoretically compared to known limb lead reversals and experimentally proven for unknown limb–chest electrode swaps using a 12-lead ECG database from 25 healthy volunteers (recordings without electrode swaps and with 5 unicolor pairs swaps, including red (right arm—C1), yellow (left arm—C2), green (left leg (LL) —C3), black (right leg (RL)—C5), all unicolor pairs). Two applications of the transformation are shown to be feasible: ‘Forward’ (simulation of reordered leads from correct leads) and ‘Inverse’ (reconstruction of correct leads from an ECG recorded with known electrode reversals). Deficiencies are found only when the ground RL electrode is swapped as this case requires guessing the unknown RL electrode potential. We suggest assuming that potential to be equal to that of the LL electrode. The ‘Forward’ transformation is important for comprehensive training platforms of humans and machines to reliably recognize simulated electrode swaps using the available resources of correctly recorded ECG databases. The ‘Inverse’ transformation can save time and costs for repeated ECG recordings by reconstructing the correct lead set if a lead swap is detected after the end of the recording. In cases when the electrode reversal is unknown but a prior correct ECG recording of the same patient is available, the ‘Inverse’ transformation is tested to detect the exact swapping of the electrodes with an accuracy of (96% to 100%).

## 1. Introduction

The routine use of the standard 12-lead electrocardiogram (ECG) for noninvasive clinical investigation of acute and chronic cardiovascular diseases makes it very important to ensure the generation of diagnostically interpretable ECG leads [1]. An essential problem in the recording of multilead ECGs is the improper placing of the electrodes on the patient’s body [2], reported to be as frequent as 0.8% and 7.5% for limb lead reversals in 12-lead ECG and Holter devices, respectively [3]; 0.4% and 4% for 12-lead ECG interchanges in clinical and intensive care settings, respectively [4]. Errors in electrode placement can lead to significant ECG changes that could confuse physicians and affect the clinical diagnosis. Studies and case reports dedicated to this problem are further grouped in the following three categories according to the affected ECG electrodes:

- *Reversals between limb electrodes* are reported to provoke deep QS complexes and inverted T waves in leads (II, III, aVF) that could be misdiagnosed as old myocardial infarctions (MI) involving the inferior heart wall [5]. Right arm (RA) and left arm (LA) interchange is associated with inverted T waves in leads (I, aVL) suggestive of lateral wall MI [6] as well as indicative of ECG features of dextrocardia [7]. RA and right leg (RL) swap results in low-amplitude QRS complexes in lead II [7,8] and all other frontal leads resembling scaled variations of lead III and changed QRS axes in the frontal plane [8]. LA and left leg (LL) reversal creates suspicions of inferior-wall MI [7]. RA and LL swap could be confused for the combined features of lateral wall MI and low atrial rhythm [7].
- *Reversals between chest electrodes* have been found to provoke erroneous diagnosis in 17% to 24% of cases involving wrongly placed C1 electrodes [9]. Generally, when another precordial lead is substituted for V1, the result is a tall R wave in V1, which could be taken as a sign of right bundle branch block, left ventricular ectopy, right ventricular hypertrophy, acute right ventricular dilation, Type A Wolff-Parkinson-White syndrome, posterior MI, hypertrophic cardiomyopathy, progressive muscular dystrophy or dextrocardia [10].
- *Reversals between limb and chest electrodes* are a possible scenario due to the matching colors of the two ECG cables [11] or the incorrect attachment of the cable connectors to the junction box of the ECG machine [12]. C2/LA (yellow) cable interchange is described in two case reports [13,14] to have produced right axis deviation and Q waves in (III, aVF), accompanied by an inverted T wave in both leads, together with a quick transition in V2 with qR complex and an inverted T wave. The ECGs are interpreted as an inferior MI with residual ischemia in [13] or recent inferior and a posterior MI [14]. Limb/precordial cable interchange has been observed to result in tall R waves in aVR, negative QRS complexes in the other five limb leads and inverted ST elevation/depression in some of the leads. Thus, inferior, anterior, and lateral MI could be erroneously diagnosed [12]. In another study [15], the authors suspect the same interchange to have resulted in ST-segment elevation in the inferior leads; however, their thesis has been impugned by [16], who have explained the wandering ST elevation with medical reasons.

Such studies indicate that special measures should be taken to ensure the correct placement of ECG electrodes, e.g., staff training has been reported to improve electrode placement by 50% [17], while combined training and technical improvements have succeeded to reduce the rate of electrode cable reversals from 4.8 % down to 1.2 % [18]. ECG changes induced by ECG cable reversals have been analyzed in a number of studies [19,20,21,22,23,24]. Methods for the automatic detection of ECG electrode reversals within the limb and precordial set have been proposed, such as:

- *Limb leads:* LA and LL reversal is indicated by P wave amplitude [25] and QRS, P-axes [26]; RA and RL interchange is detected when lead II presents as a flat line [27] or with peak-to-peak amplitude less than 185 µV [8]; LA-RA and RA-LL swaps are recognized by analysis of P and QRS frontal axes and clockwise vector loop rotation direction, R and T wave amplitudes in leads (I, II) [28]; various LA/RA/LL/RL combinations are detected by a number of analytical approaches based on the assessment of the QRS axis [29,30], together with P wave amplitudes [31], direction of P-loop inscription and/or frontal P-axis [32]; lead reconstruction using redundancy of information in eight independent leads [33]; morphological measurements of QRS, P-wave amplitudes, frontal axis and clockwise vector loop rotation, combined with redundancy features [34]; maximal and minimal QRS, T-wave amplitudes in leads (I, II, III) [35]; correlation coefficients of limb leads vs. V6 [36,37]; combining the features described in [26] and [33] for a more robust and accurate performance [36].
- *Chest leads:* Different reversal sets have been examined, such as five reversals of adjacent leads (V1/V2, V2/V3, V3/V4, V4/V5, V5/V6), analyzed by P, QRS and ST-T measurements [26] and PQ-RS amplitude distances [31]; nine reversals (five adjacent leads, V1/V3, V4/V6, V4/V5/V6/V1/V2/V3, V6/V5/V4/V3/V2/V1) are evaluated via correlations between measured and reconstructed leads [33]; seven reversals (five adjacent leads, V1/V3, V4/V6) are handled by processing of both morphology and redundancy features [34]; 15 reversals, including all possible pairwise V1–V6 swaps, have been tested in our previous study by applying analysis of inter-lead correlation coefficients [38].
- *Limb and chest leads:* Interchanges between limb and C2 precordial electrodes specific for a telemonitoring system are detected by correlation to a previously recorded ECG [39]. This early work, together with our recent publication on the unicolor electrode interchange detection [11], are the only studies dealing with recognition of reversals between limb and precordial leads.

The methods for the detection of ECG cable reversals should be designed/tested using dedicated databases. Only a few of the mentioned studies [7,19,27,35,39,40] use real ECG recordings with erroneous electrode placements, which are, however, small-sized and proprietary. Typically, reversal detection algorithms are trained and validated using databases with correctly recorded 12-lead ECGs and simulated reversals within the limb lead set [11,26,28,29,30,31,32,33,34,36,38] or the precordial lead set [11,26,31,33,34,38], where the Wilson’s central terminal (WCT) is not changed. All other reversals modifying the WCT, such as swaps between the limb and precordial electrodes, have not been simulated, although they are quite possible and should be detected due to the distorted morphology of most leads [6,8,21]. For example, the interactive web-based tool [41] for the rendering of ECG leads from body surface potential maps (BSPM) separately simulates two effects—precordial lead misplacement (by linear interpolation from neighboring BSPM leads) or limb lead interchange. However, considering that this tool uses bulky BSPM data and does not allow for electrode misplacements that change WCT potential, it has restricted application for machine learning on large arrhythmia datasets with arbitrary ECG electrode reversals.

We have not found in the literature an algebraic transformation that can simulate all possible ECG electrode reversals. This paper presents the formula of such a transformation and its application in the standard 12-lead ECG setup, computing reordered leads and the WCT potential change from the correctly recorded leads. Additionally, we show the applicability of this method for reconstructing the correct leads from an ECG recorded with known electrode reversals, as well as for detection of the exact electrodes that were swapped, provided there are at least two ECGs from the same patient.

## 2. Methods

### 2.1. Derivation of the Transformation Formula

The presented transformation formula can be used to simulate reversals between arbitrary ECG electrodes in the standard 12-lead ECG, although the final transformation formula can be extended to an arbitrary number of leads.

**Figure 1:**
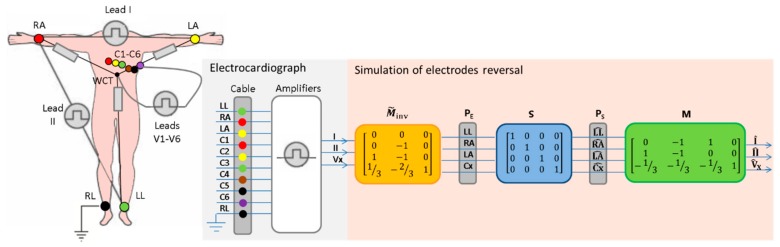
Acquisition of 12-lead ECG via a 10-electrode cable with standard IEC color coding, recording 8 independent leads I, II, V1-V6 (denoted as Vx) in the ECG device. The flow diagram shows the simulation of reversals between LL, RA, LA and one chest electrode (denoted as Cx) by conversion of the recorded leads (I, II, Vx) to reordered leads ($\hat{I},\;\hat{\mathrm{II}}$,${\hat{V}}_{X}$) using the matrix transformations ${\tilde{M}}_{\mathrm{inv}},\;S,\;M\;$. The specific example shows an identity S  matrix that corresponds to the correct order of LL, RA, LA, Cx electrodes. Other estimates of the S matrix are presented on Figure 2 and Section 4.1 in the description of different examples for electrode reversals.

According to the fundamental principles of electrocardiography [42], standard 12-lead ECG systems (Figure 1) use 10 electrodes on the LA, RA, LL, RL and 6 precordial positions (C1-C6), and acquire 8 independent signals (e.g. leads I, II, V1-V6):

(1) $$|\begin{array}{l} I=P_{E}(LA)-P_{E}(RA)\\ II=P_{E}(LL)-P_{E}(RA)\\ V_{X}=P_{E}(C_{X})-P_{WCT}=P_{E}(C_{X})-\frac{P_{E}(LL)+P_{E}(RA)+P_{E}(LA)}{3} \end{array}$$

where:

- $P_{E}$ denotes the electrical potential of the respective electrodes, also referred to as the raw electrode biopotential.
- (I, II) are the bipolar leads measuring the potential differences between limbs (LA-RA, LL-RA), forming the Einthoven’s triangle.
- $V_{X}$ represents any of the unipolar chest leads (*V1*-*V6*) measuring the potential of the chest electrodes against the reference WCT potential ($P_{WCT})$, which is defined to be the average of the RA, LA and LL electrodes.
- Note that the ground electrode placed on the RL is used for technical reasons (driven right leg) and does not have direct influence on any ECG leads.

The calculations in (1) are usually performed by the input circuits in ECG devices. As soon as an electrode swap can lead to a change in the WCT potential, it becomes difficult to imagine the changes in the standard 12-lead ECG. Therefore, the derivation and handling of the electrode potentials corresponding to the nine active ECG electrodes with respect to a common reference point is the main target of further mathematical transformations.

The basic 12-lead ECG computations (1) can also be presented using the matrix notation:

(2) [IIIVX]=[0−1101−100−13−13−131][PE(LL)PE(RA)PE(LA)PE(CX)]=M[0PE(RA)PE(LA)PE(CX)],

where M is the matrix that converts the raw electrode potentials P_E_(LL), P_E_(RA), P_E_(LA) and P_E_(Cx) into leads I, II, Vx. Formula (2) shows the setting $\left(P_{E}\left(LL\right)=0\right)$, which defines our choice that $P_{E}\left(LL\right)$ is the reference potential. This is an arbitrary choice because we can set any electrode as the reference one without changing the final outcome of our derivations. We can further simplify (2):

(3) [IIIVX]=[−110−100−13−131][PE(RA)PE(LA)PE(CX)]=MF[PE(RA)PE(LA)PE(CX)],

where $M_{F}$ is a full-rank matrix that is further inverted ($M_{F}^{-1}$) for solving of the opposite task for the conversion of leads into body electrode potentials:

(4) [PE(RA)PE(LA)PE(CX)]=MF−1[IIIVX]=[0−101−1013−231][IIIVX].

Further, $M_{F}^{-1}$ is extended so that (4) is able to reproduce the electrical potential of the left leg, using the definition $\left(P_{E}\left(LL\right)=0\right)$:

(5) [PE(LL)PE(RA)PE(LA)PE(CX)]=[000MF−1][IIIVX]=[0000−101−1013−231][IIIVX]=M˜inv[IIIVX],

where ${\tilde{M}}_{\mathrm{inv}}$ is the matrix that allows the computation of the raw body electrode potentials in the order $\left\{P_{E}\left(LL\right),\;P_{E}\left(RA\right),P_{E}\left(LA\right),\;P_{E}\left(C_{X}\right)\right\}$ using the recorded leads {I, II, Vx}. Once the body electrode potentials are known, they can be reordered to simulate an arbitrary reversal between ECG electrodes. The simulated electrode order can be algebraically described by a binary swap matrix (S), which equals an identity matrix for the correct order:

(6) $$\left[\begin{array}{c} P_{S}\left(\hat{LL}\right)\\ P_{S}\left(\hat{RA}\right)\\ P_{S}\left(\hat{LA}\right)\\ P_{S}\left(\hat{C_{X}}\right) \end{array}\right]=[\begin{array}{cccc} 1 & 0 & 0 & 0\\ 0 & 1 & 0 & 0\\ 0 & 0 & 1 & 0\\ 0 & 0 & 0 & 1 \end{array}][\begin{array}{c} P_{E}\left(LL\right)\\ P_{E}\left(RA\right)\\ P_{E}\left(LA\right)\\ P_{E}\left(C_{X}\right) \end{array}]=S[\begin{array}{c} P_{E}\left(LL\right)\\ P_{E}\left(RA\right)\\ P_{E}\left(LA\right)\\ P_{E}\left(C_{X}\right) \end{array}]$$

where $P_{S}$ denotes the simulated electrical potential at the respective electrode $\left\{\hat{LL},\;\hat{RA},\;\hat{LA},\hat{C_{X}}\right\}$. Different examples of matrix S are further given in Section 4.1 upon the description of the performed theoretical simulations of electrode reversals.

The correspondence between the leads recorded by the ECG device (I, II, Vx) and the reordered leads ($\hat{I},\;\hat{II}$,$\;{\hat{V}}_{X}$) after the simulation of ECG electrode reversals can be calculated by substituting successively (6) and (5) into (2):

(7) $$\left[\begin{array}{c} \hat{I}\\ \hat{II}\\ {\hat{V}}_{X} \end{array}\right]=M\left[\begin{array}{c} P_{S}\left(\hat{LL}\right)\\ P_{S}\left(\hat{RA}\right)\\ P_{S}\left(\hat{LA}\right)\\ P_{S}\left(\hat{C_{X}}\right) \end{array}\right]=MS{\tilde{M}}_{\mathrm{inv}}\left[\begin{array}{c} I\\ II\\ V_{X} \end{array}\right].$$

The flow diagram of (7), further denoted as ‘MSMinv’ transformation, which is presented on Figure 1, clearly indicates the embedded logic of matrix operations that have a general applicability to simulate arbitrary configurations of electrode swaps. It is just necessary to adapt the values of the matrices $M,S,\;{\tilde{M}}_{\mathrm{inv}}$ to the specific lead configuration, assuming that the derived mathematical proof shows the full set of two independent bipolar limb leads in 12-lead ECG (can be reduced) and one unipolar lead (can be deleted or extended to multiple unipolar leads by copy of the row ($C_{x})$ in $M,\;{\tilde{M}}_{\mathrm{inv}}$ and expand S accordingly).

Note that the matrices in the ‘MSMinv’ transformation (7) take into account only the potentials of the active input electrodes, excluding the grounded RL. The result of a swap of an arbitrary active electrode with RL can be, however, approximated by setting the potential of the swapped active electrode equal to the LL potential in the matrix S (6):

(8) $$P_{S}\left(swapped\;electrode\;to\;RL\right)=P_{E}\left(RL\right)\approx P_{E}\left(LL\right)$$

The assumption for equipotential legs can be considered from an anatomical perspective because the leg recording sites are sufficiently distant and similarly oriented to the heart, thus attaining the same electrical signal generated by the myocardium [24]. Generally, both (RL, LL) potentials are essentially very similar that is typically adopted in the known ECG lead transformations of rotated RL with other peripheral electrodes [7,8,21,22].

Another application of the derived mathematical transformations is the calculation of the WCT potential change due to ECG electrode swaps:

(9) $$\Delta P_{WCT}={\hat{P}}_{WCT}-P_{WCT},$$

where $P_{WCT}$ and ${\hat{P}}_{WCT}$ are the WCT potentials before and after the electrode swap, respectively. Both can be derived as a function of the recorded leads (I, II, Vx) with a reference to a zero LL potential $\left(P_{E}\left(LL\right)=0\right)$, as assumed in Equations (2) and (5):

(10) $$P_{WCT}=W\left[\begin{array}{c} P_{E}\left(LL\right)\\ P_{E}\left(RA\right)\\ P_{E}\left(LA\right)\\ P_{E}\left(C_{X}\right) \end{array}\right]=W{\tilde{M}}_{\mathrm{inv}}\left[\begin{array}{c} I\\ II\\ V_{X} \end{array}\right]$$

(11) $${\hat{P}}_{WCT}=W\left[\begin{array}{c} P_{S}\left(\hat{LL}\right)\\ P_{S}\left(\hat{RA}\right)\\ P_{S}\left(\hat{LA}\right)\\ P_{S}\left(\hat{C_{X}}\right) \end{array}\right]=WS{\tilde{M}}_{\mathrm{inv}}\left[\begin{array}{c} I\\ II\\ V_{X} \end{array}\right],$$

where $W=\left[\begin{array}{cccc} 1/3 & 1/3 & 1/3 & 0 \end{array}\right]$ is the matrix that transforms electrode potentials into WCT potential, taking the potentials for the correct electrode position $\left\{P_{E}\left(LL\right),\;P_{E}\left(RA\right),P_{E}\left(LA\right),\;P_{E}\left(C_{X}\right)\right\}\;$ from (5) and for the swapped position $\left\{P_{S}\left(\hat{LL}\right),\;P_{S}\left(\hat{RA}\right),P_{S}\left(\hat{LA}\right),\;P_{S}\left(\hat{C_{X}}\right)\right\}$ from (6) and (5). Although WCT potential is calculated only from the potentials of the three limb electrodes $\left(\hat{LL,\;}\hat{RA},\;\hat{LA}\right)$, equation (11) covers the general option for a swap between some of them and the unipolar electrode $\left(\hat{C_{X}}\right)$.

Substituting (10) and (11) in (9) gives the generalized notation of the ‘WSMinv’ transformation that is further used for estimating of the relative WCT potential change during different simulated swaps:

(12) $$\Delta P_{WCT}=\left(WS-W\right){\tilde{M}}_{\mathrm{inv}}\left[\begin{array}{c} I\\ II\\ V_{X} \end{array}\right].$$

### 2.2. Verification of ‘MSMinv’ Transformation

The correctness of the ‘MSMinv’ transformation (7) is verified by two approaches, depending on the kind of simulated ECG electrode reversals:

- *ECG electrode reversals with known lead transforms* are theoretically studied. For this purpose, the formula for computation of the reordered leads ($\hat{I},\;\hat{II}$, $\;{\hat{V}}_{X}$) is directly compared to the published lead transformations. This simple approach is applicable only to reversals between peripheral electrodes, widely analyzed in the literature [5,7,12,21,24,36,37,38,43].
- *ECG electrode reversals with unknown lead transformations* (such as reversals between limb and chest electrodes) are experimentally studied with a dedicated database (described in Section 3). For this purpose, the 8 independent leads $L_{R}=\left(I_{R},\;II_{R},\;\;V1_{R}-V6_{R}\right)$ of 2 recordings from the same person (RC, taken with correct electrode position; RS, taken with real electrode swap) are compared in three different scenarios:
  ◦ ($L_{RC}\;vs.\;L_{RS})$*: No transformation* is applied to study the lead-specific differences between recordings with correct vs. swapped electrodes.
  ◦ ($\hat{L_{RC}}\;vs.\;L_{RS})$: *Forward ‘MSMinv’ transformation* is applied on the recording with correct lead set to simulate lead swap $\left(\hat{L_{RC}}\;=MS{\tilde{M}}_{\mathrm{inv}}L_{RC}\right)\;\;$ and to study the lead-specific differences of simulated vs. recorded electrode reversals ($L_{RS})$.
  ◦ ($L_{RC}\;vs.\;\hat{L_{RS}})$: *Inverse ‘MSMinv’ transformation* is applied on the recording with reversed lead set to simulate correct electrode positions ($\hat{L_{RS}}\;=MS{\tilde{M}}_{\mathrm{inv}}L_{RS})\;$ and to study the lead-specific differences of simulated vs. recorded, correctly placed electrodes ($L_{RC})$.

The lead-specific differences in each of the above 3 scenarios (denoted as $\tilde{L_{RC}}\;vs.\;\tilde{L_{RS}})$ are estimated for the average beat $(BEAT_{i}),\;\;$ indexed within a window of 500ms ($i=QRS_{f}-150ms\;to\;QRS_{f}+350ms$, where $QRS_{f}$ denotes the QRS fiducial point), using three quantitative measures:

◦ Root-mean-square error:
  (13) $$RMS\;Error=\sqrt{\frac{1}{500ms*Fs}\sum {\left(BEAT_{i}\left(\tilde{L_{RC}}\right)-BEAT_{i}\left(\tilde{L_{RS}}\right)\right)}^{2}},$$
  where Fs denotes the sampling frequency of the average beat.
◦ Peak error:
  (14) $$Peak\;Error=\max(\left|BEAT_{i}\left(\tilde{L_{RC}}\right)-BEAT_{i}\left(\tilde{L_{RS}}\right)\right|.$$
◦ Correlation coefficient:
  (15) $$CorCoef=\frac{\sum BEAT_{i}\left(\tilde{L_{RC}}\right).BEAT_{i}\left(\tilde{L_{RS}}\right)}{\sqrt{\sum BEAT_{i}^{2}\left(\tilde{L_{RC}}\right).\sum BEAT_{i}^{2}\left(\tilde{L_{RS}}\right).}}$$

Statistical results of all quantitative measurements over the whole ECG database are reported as a mean value and standard deviation (std). The level of significant differences between different scenarios is measured with paired Student’s t-test and one-tailed *p*-value < 0.05.

## 3. Database

The database used for verification of the ‘MSMinv’ transformation contains 10s recordings of standard 12-lead resting ECGs taken from 25 volunteers with no history of heart diseases—gender: 28% (male), age: 49 ± 11 years (mean value ± standard deviation), 28–67 years (range). The ECGs are acquired via a 10-electrode cable with standard IEC color coding [44]. Six ECG recordings per subject are collected, applying prospective electrode cable reversals at the time of the recording, including:

- Correct positions of the electrodes (no electrode is swapped);
- Swap of red electrodes (RA-C1);
- Swap of yellow electrodes (LA-C2);
- Swap of green electrodes (LL-C3);
- Swap of black electrodes (RL-C5);
- Swap of all unicolor electrodes (RA-C1, LA-C2, LL-C3, RL-C5).

The ECG signals are recorded at 1 kHz sampling rate, 1 µV resolution, and pre-filtered in a bandwidth (0.5 to 25 Hz). Each 10s ECG recording is processed by a commercial ECG measurement and interpretation module (ETM, Schiller AG, Switzerland) for the extraction of a 12-lead average beat [45]. The average beats are commonly used for the measurement of ECG features with diagnostic precision because they provide higher signal-to-noise ratio and are more robust to respiration induced morphology changes than the single beats.

## 4. Results and Discussion

### 4.1. Theoretical Simulations of Electrode Reversals

**Figure 2:**
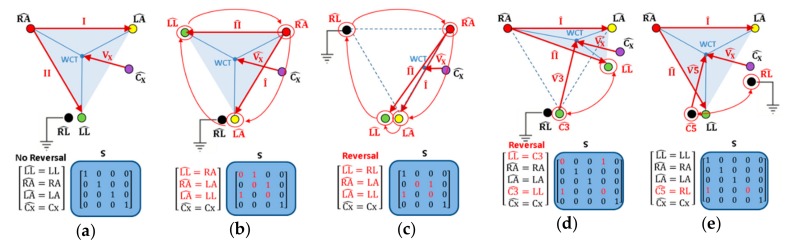
Classic depiction of the Einthoven’s triangle, WCT and lead vectors (I, II, Vx), projected in the frontal plane for correct electrode position (**a**) and their displacement ($\hat{I},\;\hat{\mathrm{II}}$,${\hat{V}}_{X}$) in case of four types of limb electrode reversals: (**b**) CW rotation of 3 active limb electrodes ($\hat{\mathrm{RA}}\;$→$\hat{\mathrm{LA}}$→$\hat{\mathrm{LL}}$→$\hat{\mathrm{RA}}$); (**c**) CW rotation of 4 limb electrodes, including RL ($\hat{\mathrm{RL}}$→$\hat{\mathrm{RA}}$→$\hat{\mathrm{LA}}$→$\hat{\mathrm{LL}}$→$\hat{\mathrm{RL}}$); (**d**) swap of an active limb electrode and chest electrode, illustrated for the green couple ($\hat{\mathrm{LL}}$↔$\hat{C3}$); (**e**) swap of the grounded and chest electrode, illustrated for the black couple ($\hat{\mathrm{RL}}$↔$\hat{C5}$). The text with red font color highlights the electrodes in wrong geometrical positions, and the swap matrix entries different from the identity matrix in (**a**).

This section simulates three major types of ECG electrode reversals (reversals of peripheral electrodes involving RL; not involving RL; reversals of peripheral and chest electrodes ), applying ‘WSMinv’ transformation (12) for the calculation of WCT potential change (Table 1) and ‘MSMinv’ transformation (7) for the calculation of the reordered leads (Table 2, Table 3 and Table 4). Several general examples are shown on Figure 2. Details will be further discussed in Section 4.1.1, Section 4.1.2 and Section 4.1.3

#### 4.1.1. Reversals of Peripheral Electrodes Not Involving RL

All 5 possible rotations of the 3 active limb electrodes ($\hat{LL},\;\hat{RA},\;\hat{LA}$) are simulated (Table 2) and none of them is related to the displacement of WCT, as illustrated in the example of clockwise (CW) electrode rotation (Figure 2b), where the Einthoven’s triangle remains geometrically unaffected. This is theoretically proven by the ‘WSMinv’ transformation (12), where all swap matrices S (Table 2) are consequently applied, and zero WCT potential differences to the correct electrode position ($\Delta P_{WCT}=0$) are detected (Table 1). The rearranged limb leads ($\hat{I},\;\hat{II}$) obtained by the ‘MSMinv’ transformation (Table 2) match the expressions in other studies, applying geometrical perspectives between leads (I, II, III) [5,12,21,24,36,37,38]. Furthermore, the unipolar chest lead $\;{\hat{V}}_{X}$ is unchanged for all simulated swap matrices S that corresponds to the real case scenario of unchanged WCT (Figure 2a,b).

#### 4.1.2. Reversals of Peripheral Electrodes Involving RL

Seven rotations of the 4 limb electrodes ($\hat{LL},\;\hat{RA},\;\hat{LA},\hat{RL}$) are simulated (Table 3) and normally they should be related to the displacement of WCT, as illustrated in the example (Figure 2c), where the Einthoven’s triangle is transformed to a thin ‘slice’. 

Table 1 shows that all electrode reversals present WCT potential change, depending only on the position of the neutral electrode RL, such that:

- RL is in the position of RA: $\Delta P_{WCT}=1/3II$
- RL is in the position of LA: $\Delta P_{WCT}=-1/3I+1/3II$
- RL is in the position of LL: $\Delta P_{WCT}=0$, where $P_{WCT}$ is equal to the correct electrode placement.

These results and the ‘MSMinv’ transformation (Table 3) are obtained with the general approximation for equipotential legs that is coded in the swap matrix S with an entry of ‘1’ in the position of LL (first column) for both cases: $\left\{\hat{X}=LL,\;\;\hat{X}=RL\right\}$, whereas $\hat{X}$ denotes an arbitrary ECG electrode. Thus, all electrode reversals leading to a ‘sliced’ Einthoven’s triangle with two tips on both legs appear with two ‘1’ entries in the first column of matrix S (Table 3), while ‘1’ is deficient in the second (RA) or third (LA) columns (equal to ‘0’). Only two reversals (RL-LL and the counter-clockwise (CCW) rotation with RL) have ‘1’ entries in each of the first three columns of matrix S (corresponding to the three active limb electrodes in the Einthoven’s triangle tips). Just for them, the WCT potential is correctly found to be unchanged (Table 1), although the reordered limb leads ($\hat{I},\;\hat{II}$) in CCW rotation appear to be different (Table 3). Generally, all simulated reversals show that ($\hat{I},\;\hat{II}$) leads, resulting from the ‘MSMinv’ transformation, are matching the expressions in other studies that have been derived by geometrical analysis of the leads, assuming “0” [6,12,19,21,22,24,35,36] or “near zero” signal <100 μV [5,7,8,38] for the lead between the active electrodes on both legs.

Considering the chest lead$\;V_{X}$, we find a correspondence between ‘MSMinv’ and ‘WSMinv’ transformations so that the reordered lead ${\hat{V}}_{X}=\;V_{X}-\Delta P_{WCT}$ (Table 3) is corrected exactly with the term $\Delta P_{WCT}$ (Table 1). Generally, no chest lead $V_{X}$ is considered in case of limb leads reversals, so we cannot compare the derived$\;{\hat{V}}_{X}$ expressions to published studies. We can justify the unchanged ${\hat{V}}_{X}$ in both aforementioned reversals with WCT not being displaced (RL-LL and CCW rotation), as well as ${\hat{V}}_{X}=V_{X}-1/3II$ for the RL-RA reversal. This reversal has been analytically described in Haisty et al. [8], who explained that WCT potential difference is equal to one third of the difference between RA and RL potentials that approximates one third of the standard lead II.

#### 4.1.3. Reversals of Chest and Peripheral Electrodes

Five swaps are simulated (Table 4) involving the matching-color electrode pairs in the peripheral and precordial cables (IEC color coding standard [44]), i.e., red (RA-C1), yellow (LA-C2), green (LL-C3), black (RL-C5) and all pairs (RA-C1, LA-C2, LL-C3, RL-C5). 

Figure 2d,e illustrates the two principal types of reversals where the unipolar chest electrode is swapped either with an active limb electrode (WCT is displaced in Figure 2d) or with the grounded electrode (WCT is not displaced in Figure 2e). In both cases, we highlight two types of unipolar leads:

- $\hat{Vx}$ for the precordial electrodes $\hat{Cx}$, which keep their position unchanged on the chest;
- $\hat{Vn}$ for the precordial electrodes $\hat{Cn}$, where n = 1,2,3,5 is substituting the chest electrode number, which changes its position to some of the limbs.

The calculation of two different unipolar leads is achieved by an extension of the swap matrix S (4 × 4) to (5 × 5) to include 3 limb electrodes and 2 chest electrodes ($\hat{LL},\;\hat{RA},\;\hat{LA},\hat{Cn},\;\hat{Cx}$), as shown in Table 4 and Figure 2d,e. The last row of Table 4 presents the most complex example for simulation of swaps between all unicolor electrode pairs, which requires the use of a swap matrix S (9 × 9), configured for the full set of electrodes ($\hat{LL},\;\hat{RA},\;\hat{LA},\hat{C1}-\hat{C6}$). All expressions of the rearranged leads ($\hat{I},\;\hat{II},\hat{Vx},\;\hat{Vn})$ in Table 4 are further verified in the experimental study of Section 4.2 because they have not been investigated in any other study. The respective WCT potential changes (Table 1) cannot be compared to examples in the literature either. We can only justify the result for the RL-C5 reversal, which corresponds to non-displaced WCT, exactly as shown in the example (Figure 2e).

### 4.2. Experimental Verification of Simulated Swaps Between Unicolor Chest and Peripheral Electrodes

The experimental study is used to verify the ‘MSMinv’ transformation for simulation of reversals between unicolor chest and peripheral electrodes (using the expressions in Table 4), according to the concept in Section 2.2 (*ECG electrode reversals with unknown lead transformations*). For this purpose, all ECG recordings in the database are analyzed and the three measurements (RMS Error, Peak Error, CorCoef) are calculated to estimate the average beat waveform differences in 3 scenarios (Table 5):

- *No transformation*, showing the largest differences between correct vs. swapped electrode recordings for all leads because WCT is considerably displaced in most chest-limb reversals (except RL-C5). We note the greatest mean value differences for the unipolar lead with a chest electrode placed on the limbs:
  ◦ V1 (120 μV, 529 μV, 0.832) for RA-C1,
  ◦ V2 (246 μV, 1121 μV, 0.456) for LA-C2,
  ◦ V3 (206 μV, 868 μV, 0.512) for LL-C3,
  ◦ V5 (131 μV, 639 μV, 0.785) for RL-C5,
  ◦ V1-V3 (225-289 μV, 967-1235 μV, 0.365-0.652) for all unicolor pairs.
- *Forward ‘MSMinv’ transformation*, simulating electrode reversals which have significantly reduced differences when compared to the recordings with really swapped electrodes (*p* < 0.05). We measure mean values (RMS Error, Peak Error, CorCoef) in the range (<20 μV, <60 μV, ≥0.995), assuming they represent negligible average beat differences mainly due to rhythm variation and signal acquisition noises in the compared recordings. We have noticed one exception for both reversals involving RL (RL-C5, all unicolor pairs), where the Forward ‘MSMinv’ transformation introduces a slight error in the calculation of the swapped lead V5 (≤26 μV, ≤104 μV, ≥0.986), assuming the C5 potential to be equal to LL, while C5 is placed on the RL (approximation error from the equipotential legs).
- *Inverse ‘MSMinv’ transformation*, recovering the correct electrode order, which has significantly reduced differences when compared to the recordings with really correct electrodes (*p* < 0.05), estimated within the above outlined range of negligible errors (<20 μV, <60 μV, ≥0.995). We have again found an exception for both reversals involving RL (RL-C5, all unicolor pairs), where the Inverse ‘MSMinv’ transformation fails to reconstruct the correct lead V5 (<142 μV, <660 μV, ≥0.792) from a recording with RL electrode in the position of C5 electrode. As soon as RL stops being an input to the ECG device, the potential of V5 electrode is lost and not reproduced by any active electrode in the swap matrix S (Table 4, all S entries are equal to ‘0’ for the column, corresponding to C5).

**Figure 3:**
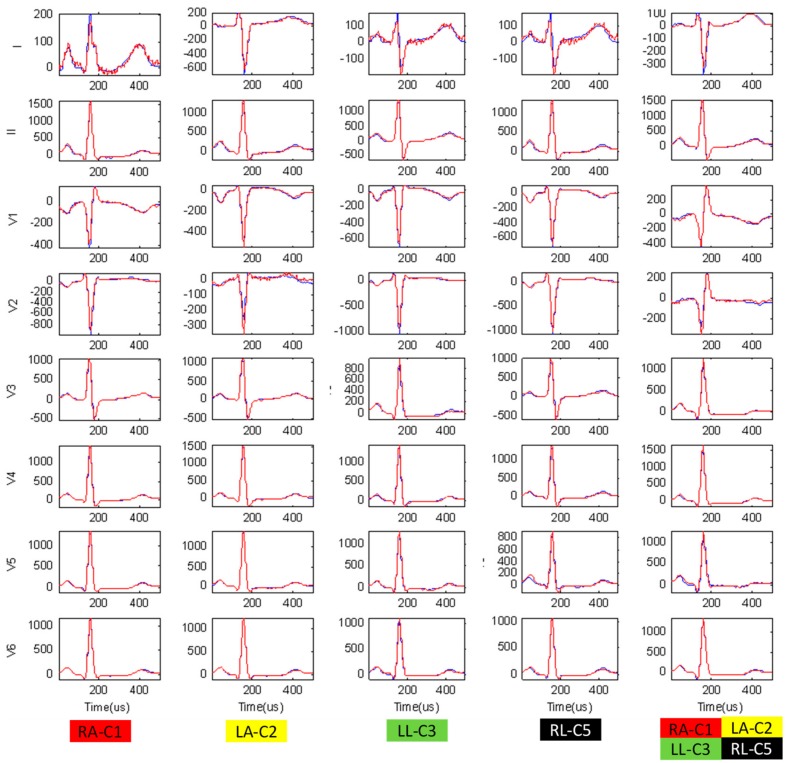
The average beat of 8 independent leads (I, II, V1-V6) taken from the same subject in two scenarios: (1) Red trace: Recorded ECG with swapped unicolor electrodes; (2) Blue trace: Simulated ECG with swapped unicolor electrodes, applying Forward ‘MSMinv’ transformation on the ECG recorded with the correct lead set.

Figure 3 illustrates the average ECG beats in leads (I, II, V1-V6) recorded with all types of swapped unicolor chest and peripheral electrodes. This ECG trace is almost fully overlapping with the simulated electrode reversals from the ECG raw data with correct electrode positions, applying Forward ‘MSMinv’ transformation. This once again validates the derived algebraic transformations in Table 4, which are able to exactly reproduce the diversity of lead-specific morphologies (amplitudes, polarities and durations) that each swap introduces to any lead via change of its electrode position and/or WCT potential.

### 4.3. Application of the ‘MSMinv’ Transformation for Automatic Detection of the Exact ECG Electrode Reversals

We further show an important practical application of the Inverse ‘MSMinv’ transformation for the detection of the exact reversals between ECG electrodes. This application is relevant to the case when two ECG recordings in a patient are available: 

(1) an ECG recording with correct lead set ($L_{RC})$; 

(2) an ECG recording with an unknown/suspected lead swap $\left(L_{RS}\right)$. 

The ‘MSMinv’ transformation is applied to the swapped ECG recording, iteratively simulating all possible permutations of the nine active ECG electrodes (i = 9! = 362 880):

(16) $$\hat{L_{RS}}\left(i\right)=MSi{\tilde{M}}_{\mathrm{inv}}L_{RS}$$

The swap matrix S**_i_**, which produces the lead set reconstruction $\hat{L_{RS}}\left(i\right)$ with the minimal difference d(·) to the correct lead set can indicate the true lead swap (TLS):

(17) $$TLS=\;\underset{1\leq i\leq 9!}{arg\;min}\left(d\left(\hat{L_{RS}}\left(i\right)-L_{RC}\right)\right)$$

We test this swap detection criterion using our ECG database under the following conditions:

- All 25 patients are considered, comparing the available pairs of recordings ($L_{RC},\;L_{RS}$ ) per patient, where $L_{RC}$ is the recording without electrode reversals, and $L_{RS}$ represents one of the recorded 3 lead swaps (RA-C1, LA-C2, LL-C3), not involving the RL.
- The minimal difference rule (17) is applied on the average beat of each recording.
- The minimal difference rule (17) is evaluated with 3 distance metrics d(·) – min(RMS Error) (Equation (13)), min(Peak Error) (Equation (14)), max(CorCoef) (Equation (15)).
- The accuracy for detection of the TLS is evaluated as the true positive (TP) rate, considering the tested population of N subjects:
  (18) $$\mathrm{Accuracy}=\overset{N}{\underset{p=1}{\sum }}\frac{{\mathrm{TP}}_{p}}{N},\;$$
  $$\mathrm{where}\;TP=f\left(x\right)=\{\begin{array}{c} 1,\;\;correct\;lead\;swap\;detected\\ 0,\;\;otherwise \end{array}$$

Table 6 presents the experimental verification of the Inverse ‘MSMinv’ transformation, applied for automatic detection of the exact ECG electrode reversals. We have achieved 100% accuracy in the detection of (RA-C1) and (LL-C3) reversals, and 96% for the detection of (LA-C2) reversal when the decision rule (17) uses a minimal difference metric equal to the minimal RMS Error or the maximal correlation between the reconstructed swap and the correct recording. All false negatives are observed to present TLS with either the 2nd or the 3rd ranked minimal differences, while the decision rule (17) is presently adjusted to simply report the exact lead swap with the 1st ranked minimal difference. For example, the lowest LA-C2 accuracy is due to one case, where the true LA-C2 is 2nd ranked, while the true LA-C2 in combination with RA-C1 is 1st ranked. Obviously, the detection of the exact electrode swap among many electrodes with close geometrical positions might include a portion of uncertainty due to indistinguishable geometrical perspectives of the heart vector in neighboring leads. Further updates of the decision rule (17) could potentially improve the accuracy for TLS detection, e.g., by majority voting of many distance metrics after exclusion of those with the lowest accuracy (i.e., the peak Error with 88% to 96%).

The presented rules are easily applicable in a warning system that can alert the medical staff to check the electrodes suggested for reversal within the first seconds of 12-lead ECG acquisition. Principally, the analysis is not fixed exactly to 10 s but can take a single beat or an average beat over ECGs with an arbitrary duration. It is important to note that the results reported in this section represent the best-case results, because they are based on resting ECG recordings that were taken within a short window of time and with exactly the same electrode positions (only the electrode cables were swapped). In a clinically relevant application, the ECGs under question will most likely have a substantially different recording date (leading to possible physiological changes in the ECG), and they will most likely be recorded with slightly different electrode positions. Although the average beat pattern of the same individual has been shown to have a long-term stability independent of recording sessions and physiological factors (age, gender, heart rate) [46,47], the reliable clinical application should always consider a final approval of the alarmed electrode reversals by the medical staff and electrode correction at the time of the recording.

## 5. Conclusions

This study derives a novel algebraic transformation which converts the standard 12-lead ECG leads from a correct to reordered set in case of arbitrary reversals between ECG electrodes. The formula of the ‘MSMinv’ transformation (7) is generalized to the calculation of all kinds of electrode reversals, giving the exact lead reordering for:

- Limb electrode swaps that have been previously drawn from anatomical perspectives (Table 2 and Table 3);
- Limb–chest electrode swaps with WCT potential change which, to the best of our knowledge, have never been simulated in the literature (Table 4). The formulas are exhibited for the most probable reversals of unicolor electrode pairs in the peripheral and precordial ECG cables.

The validity of the ‘MSMinv’ transformation is proven in our experimental study for two bilateral applications with a certain practical significance:

- *The ‘Forward’ application* computing the reordered lead set from an ECG recorded with correctly placed electrodes is important for educational purposes of both humans and machines to reliably recognize and warn of electrode swaps before potentially erroneous diagnostic interpretation has been made. In this respect, ‘MSMinv’ transformation is applicable to the available immense databases of correctly recorded ECGs (from normal and abnormal heart conditions) for reproducing the vast diversity of distorted ECG leads that can be observed in arbitrary swaps within the limb and precordial electrode set (such as the examples in Figure 3). This is an indispensable tool for the comprehensive training platforms to visualize and study the effects of electrode swaps (e.g. online cardiology courses for physicians, researchers, and instructors) or software design platforms (training the automatic detection algorithms) on abundant ECG electrode reversals.
- *The ‘Inverse’ application* reconstructing the correct lead set from an ECG recorded with known electrode reversals can save time and the cost of having to repeat ECG recordings in case of follow-up detection of electrode reversals and error-screening of clinical databases. The possibility for straightforward visualization of the correct lead set and its interpretation can easily uncover diagnostic errors or answer dilemmas for suspected or mistrusted electrode swaps (such as the dispute between [15] and [16]). In the general case when the electrode reversal is unknown but a prior correct ECG recording of the same patient is available, the Inverse ‘MSMinv’ transformation is able to solve the non-trivial task of detecting which electrodes have been exactly swapped. For this purpose, all possible swapped reconstructions can be simulated to ultimately determine the one which yields the minimal RMS difference or the highest correlation with the known recording. We have achieved a detection accuracy of 96% to 100% for this simple criterion, which has been tested with available recordings of three electrode swaps (RA-C1, LA-C2, LL-C3, not involving the RL) from 25 healthy persons. One should consider, however, the limitations for the validation of such an application which should be tested against the functional and physiological ECG instability between the two ECG sessions (e.g., the criterion validated on a specific population with “normal” ECG during rest could fail on cardiac patients or patients under environmental stress with lead-wise ECG morphology change). As this work is focused only on the problem of simulating all possible ECG cable reversals, the design and validation of criteria for a self-consistency check for detection of the ECG cable reversals is an extensive problem for a future study.

Another important derivation of the present study is the ‘WSMinv’ transformation (12), which computes WCT potential change for all kinds of electrode reversals. It quantifies the common distortion that all unipolar leads would meet while their reference potential is changed, regardless of whether or not they maintain their correct position. Table 1 shows that WCT distortion is proportional to leads I, II, and the displaced unipolar leads (V1-V3 in the simulation) with fractions, which are hardly envisaged without the derived transformation. The overall influence of WCT change on all unipolar leads is also reproduced by the ‘MSMinv’ transformation in Table 3 and Table 4.

This study provides the means for calculation of the potentials of the ECG electrodes and WCT toward a common reference potential using Equations (5) and (10), respectively. These mathematical transformations can be applied to any standard 12-lead ECG for deriving the ‘true’ unipolar ECG leads and WCT measurements that have recently been experimentally acquired via special ECG machines [43,47]. The experimental studies have considered the position of the reference potential on the right leg, while this study shows the values of the matrix ${\tilde{M}}_{\mathrm{inv}}$ for the reference left leg leading to the same global observations (WCT measured with respect to the left leg does not have a stable voltage and is correlated to the limb leads (I, II), exhibiting all ECG waves). Although both legs have essentially insignificant potential difference, its value is the only unknown and approximated to be a zero measurement while applying our transformation equations.

## Figures and Tables

**Table 1:** Calculation of the WCT potential change from the recorded leads via the ‘WSMinv’ transformation (12), applying the swap matrices S in Table 2, Table 3 and Table 4.

Reversed Electrodes		ΔP_WCT_
Reversals of peripheral electrodes not involving RL	$\hat{\mathrm{RA}}$↔$\hat{\mathrm{LA}}$	0*
	$\hat{\mathrm{RA}}$↔$\hat{\mathrm{LL}}$	0*
	$\hat{\mathrm{LA}}$↔$\hat{\mathrm{LL}}$	0*
	CW rotation $\hat{\mathrm{RA}}$→$\hat{\mathrm{LA}}$→$\hat{\mathrm{LL}}$→$\hat{\mathrm{RA}}$	0*
	CCW rotation $\hat{\mathrm{RA}}$→$\hat{\mathrm{LL}}$→$\hat{\mathrm{LA}}$→$\hat{\mathrm{RA}}$	0*
Reversals of peripheral electrodes involving RL	$\hat{\mathrm{RL}}$↔$\hat{\mathrm{RA}}$	1/3II
	$\hat{\mathrm{RL}}$↔$\hat{\mathrm{LA}}$	1/3(II−I)
	$\hat{\mathrm{RL}}$↔$\hat{\mathrm{LL}}$	0 *
	CW rotation with RL $\hat{\mathrm{RL}}$→$\hat{\mathrm{RA}}$→$\hat{\mathrm{LA}}$→$\hat{\mathrm{LL}}$→$\hat{\mathrm{RL}}$	1/3II
	CCW rotation with RL $\hat{\mathrm{RL}}$→$\hat{\mathrm{LL}}$→$\hat{\mathrm{LA}}$→$\hat{\mathrm{RA}}$→$\hat{\mathrm{RL}}$	0 *
	Bilateral arm–leg rotation $\hat{\mathrm{RL}}$↔$\hat{\mathrm{RA}}$, $\hat{\mathrm{LA}}$↔$\hat{\mathrm{LL}}$	1/3II
	Cross rotation $\hat{\mathrm{RL}}$→$\hat{\mathrm{RA}}$→$\hat{\mathrm{LL}}$→$\hat{\mathrm{LA}}$→$\hat{\mathrm{RL}}$	1/3(II−I)
Reversals of unicolor peripheral and chest electrodes	Red electrodes $\hat{\mathrm{RA}}$↔$\hat{C1}$	$1/9I+1/9II+1/3V_{1}$
	Yellow electrodes $\hat{\mathrm{LA}}$↔$\hat{C2}$	$-2/9I+1/9II+1/3V_{2}$
	Green electrodes $\hat{\mathrm{LL}}$↔$\hat{C3}$	$1/9I-2/9II+1/3V_{3}$
	Black electrodes $\hat{\mathrm{RL}}$↔$\hat{C5}$	0*
	All unicolor electrodes $\hat{\mathrm{RA}}$↔$\hat{C1}$, $\hat{\mathrm{LA}}$↔$\hat{C2}$, $\hat{\mathrm{LL}}$↔$\hat{C3}$, $\hat{\mathrm{RL}}$↔$\hat{C5}$	$1/3(V_{1}+V_{2}+V_{3})$

*Note:* For comprehension purposes of various electrode combinations, the reversed electrodes are depicted with their respective colors according to the IEC color coding [44]. * ΔP_WCT_ = 0 corresponds to the correct position of the active limb electrodes ($\hat{LL},\;\hat{RA},\;\hat{LA}$).

**Table 2:** Reversals of peripheral electrodes not involving RL.

Reversed Electrodes	S	$MS{\tilde{M}}_{inv}$	Reordered Leads
$\hat{\mathrm{RA}}$↔$\hat{\mathrm{LA}}$			
$\left[\begin{array}{c} \hat{LL}=LL\\ \hat{RA}=LA\\ \hat{LA}=RA\\ \hat{C_{X}}=C_{X} \end{array}\right]$	$\left[\begin{array}{cccc} 1 & 0 & 0 & 0\\ 0 & 0 & 1 & 0\\ 0 & 1 & 0 & 0\\ 0 & 0 & 0 & 1 \end{array}\right]$	$\left[\begin{array}{ccc} -1 & 0 & 0\\ -1 & 1 & 0\\ 0 & 0 & 1 \end{array}\right]$	$\hat{I}=-I\;$ $\hat{II}=-I+II=III$ ${\hat{V}}_{X}=V_{X}$
$\hat{\mathrm{RA}}$↔$\hat{\mathrm{LL}}$			
$\left[\begin{array}{c} \hat{LL}=RA\\ \hat{RA}=LL\\ \hat{LA}=LA\\ \hat{C_{X}}=C_{X} \end{array}\right]$	$\left[\begin{array}{cccc} 0 & 1 & 0 & 0\\ 1 & 0 & 0 & 0\\ 0 & 0 & 1 & 0\\ 0 & 0 & 0 & 1 \end{array}\right]$	$\left[\begin{array}{ccc} 1 & -1 & 0\\ 0 & -1 & 0\\ 0 & 0 & 1 \end{array}\right]$	$\hat{I}=I-II=-III\;$ $\hat{II}=-II$ ${\hat{V}}_{X}=V_{X}$
$\hat{\mathrm{LA}}$↔$\hat{\mathrm{LL}}$			
$\left[\begin{array}{c} \hat{LL}=LA\\ \hat{RA}=RA\\ \hat{LA}=LL\\ \hat{C_{X}}=C_{X} \end{array}\right]$	$\left[\begin{array}{cccc} 0 & 0 & 1 & 0\\ 0 & 1 & 0 & 0\\ 1 & 0 & 0 & 0\\ 0 & 0 & 0 & 1 \end{array}\right]$	$\left[\begin{array}{ccc} 0 & 1 & 0\\ 1 & 0 & 0\\ 0 & 0 & 1 \end{array}\right]$	$\hat{I}=II\;$ $\hat{II}=I$ ${\hat{V}}_{X}=V_{X}$
CW rotation $\hat{\mathrm{RA}}$→$\hat{\mathrm{LA}}$→$\hat{\mathrm{LL}}$→$\hat{\mathrm{RA}}$			
$\left[\begin{array}{c} \hat{LL}=RA\\ \hat{RA}=LA\\ \hat{LA}=LL\\ \hat{C_{X}}=C_{X} \end{array}\right]$	$\left[\begin{array}{cccc} 0 & 1 & 0 & 0\\ 0 & 0 & 1 & 0\\ 1 & 0 & 0 & 0\\ 0 & 0 & 0 & 1 \end{array}\right]$	$\left[\begin{array}{ccc} -1 & 1 & 0\\ -1 & 0 & 0\\ 0 & 0 & 1 \end{array}\right]$	$\hat{I}=-I+II=III\;$ $\hat{II}=-I$ ${\hat{V}}_{X}=V_{X}$
CCWrotation $\hat{\mathrm{RA}}$→$\hat{\mathrm{LL}}$→$\hat{\mathrm{LA}}$→$\hat{\mathrm{RA}}$			
$\left[\begin{array}{c} \hat{LL}=LA\\ \hat{RA}=LL\\ \hat{LA}=RA\\ \hat{C_{X}}=C_{X} \end{array}\right]$	$\left[\begin{array}{cccc} 0 & 0 & 1 & 0\\ 1 & 0 & 0 & 0\\ 0 & 1 & 0 & 0\\ 0 & 0 & 0 & 1 \end{array}\right]$	$\left[\begin{array}{ccc} 0 & -1 & 0\\ 1 & -1 & 0\\ 0 & 0 & 1 \end{array}\right]$	$\hat{I}=-II\;$ $\hat{II}=I-II=-III$ ${\hat{V}}_{X}=V_{X}$

*Note:* For comprehension purposes of various electrode combinations, the reversed electrodes are depicted with their respective colors according to the IEC color coding [44], *- 1st column:* The simulated placement of ECG electrodes $\left\{\hat{LL},\;\hat{RA},\;\hat{LA},\hat{C_{X}}\right\}$ referring to their geometrical positions $\left\{LL,\;RA,LA,\;C_{X}\right\}$. The electrodes which are assumed to be in the wrong geometrical positions are indicated with red font color as being reversed. *- 2nd column:* The values of the swap matrix S, where red font colored entries indicate the difference to the identity matrix as defined in (6) for the correct electrode placement. *- 3rd column:* The result of matrix multiplication $MS{\tilde{M}}_{\mathrm{inv}}$, considering M and ${\tilde{M}}_{\mathrm{inv}}$ equal to their definitions in (2) and (5), respectively. *- 4th column:* The formula for calculation of the reordered leads ($\hat{I},\;\hat{II,\;}\;{\hat{V}}_{X}$) using the recorded leads (I, II, Vx)  that is obtained after substituting $MS{\tilde{M}}_{\mathrm{inv}}$ in (7). For simplification, the substitution (III=II−I) is applied in some formulas.

**Table 3:** Reversals of peripheral electrodes involving RL.

Reversed Electrodes	S	$MS{\tilde{M}}_{inv}$	Reordered Leads
$\hat{\mathrm{RL}}$↔$\hat{\mathrm{RA}}$			
$\left[\begin{array}{c} \hat{LL}=LL\\ \hat{RA}=RL\\ \hat{LA}=RA\\ \hat{C_{X}}=C_{X} \end{array}\right]$	$\left[\begin{array}{cccc} 1 & 0 & 0 & 0\\ 1 & 0 & 0 & 0\\ 0 & 0 & 1 & 0\\ 0 & 0 & 0 & 1 \end{array}\right]$	$\left[\begin{array}{ccc} 1 & -1 & 0\\ 0 & 0 & 0\\ 0 & -1/3 & 1 \end{array}\right]$	$\hat{I}=I\;-II=-III$ $\hat{II}=0$ ${\hat{V}}_{X}=V_{X}-1/3II$
$\hat{\mathrm{RL}}$↔$\hat{\mathrm{LA}}$			
$\left[\begin{array}{c} \hat{LL}=LL\\ \hat{RA}=RA\\ \hat{LA}=RL\\ \hat{C_{X}}=C_{X} \end{array}\right]$	$\left[\begin{array}{cccc} 1 & 0 & 0 & 0\\ 0 & 1 & 0 & 0\\ 1 & 0 & 0 & 0\\ 0 & 0 & 0 & 1 \end{array}\right]$	$\left[\begin{array}{ccc} 0 & 1 & 0\\ 0 & 1 & 0\\ 1/3 & -1/3 & 1 \end{array}\right]$	$\hat{I}=II$ $\hat{II}=II$ ${\hat{V}}_{X}=V_{X}-1/3\left(II-I\right)$
$\hat{\mathrm{RL}}$↔$\hat{\mathrm{LL}}$			
$\left[\begin{array}{c} \hat{LL}=RL\\ \hat{RA}=RA\\ \hat{LA}=LA\\ \hat{C_{X}}=C_{X} \end{array}\right]$	$\left[\begin{array}{cccc} 1 & 0 & 0 & 0\\ 0 & 1 & 0 & 0\\ 0 & 0 & 1 & 0\\ 0 & 0 & 0 & 1 \end{array}\right]$	$\left[\begin{array}{ccc} 1 & 0 & 0\\ 0 & 1 & 0\\ 0 & 0 & 1 \end{array}\right]$	$\hat{I}=I$ $\hat{II}=II$ ${\hat{V}}_{X}=V_{X}$
CW rotation with RL $\hat{\mathrm{RL}}$→$\hat{\mathrm{RA}}$→$\hat{\mathrm{LA}}$→$\hat{\mathrm{LL}}$→$\hat{\mathrm{RL}}$			
$\left[\begin{array}{c} \hat{LL}=RL\\ \hat{RA}=LA\\ \hat{LA}=LL\\ \hat{C_{X}}=C_{X} \end{array}\right]$	$\left[\begin{array}{cccc} 1 & 0 & 0 & 0\\ 0 & 0 & 1 & 0\\ 1 & 0 & 0 & 0\\ 0 & 0 & 0 & 1 \end{array}\right]$	$\left[\begin{array}{ccc} -1 & 1 & 0\\ -1 & 1 & 0\\ 0 & -1/3 & 1 \end{array}\right]$	$\hat{I}=-I+II=III\;$ $\hat{II}=-I+II=III\;$ ${\hat{V}}_{X}=V_{X}-1/3II$
CCW rotation with RL $\hat{\mathrm{RL}}$→$\hat{\mathrm{LL}}$→$\hat{\mathrm{LA}}$→$\hat{\mathrm{RA}}$→$\hat{\mathrm{RL}}$			
$\left[\begin{array}{c} \hat{LL}=LA\\ \hat{RA}=RL\\ \hat{LA}=RA\\ \hat{C_{X}}=C_{X} \end{array}\right]$	$\left[\begin{array}{cccc} 0 & 0 & 1 & 0\\ 1 & 0 & 0 & 0\\ 0 & 1 & 0 & 0\\ 0 & 0 & 0 & 1 \end{array}\right]$	$\left[\begin{array}{ccc} 0 & -1 & 0\\ 1 & -1 & 0\\ 0 & 0 & 1 \end{array}\right]$	$\hat{I}=-II\;$ $\hat{II}=I-II=-III$ ${\hat{V}}_{X}=V_{X}$
Bilateral arm–leg rotation $\hat{\mathrm{RL}}$↔$\hat{\mathrm{RA}}$, $\hat{\mathrm{LA}}$↔$\hat{\mathrm{LL}}$			
$\left[\begin{array}{c} \hat{LL}=LA\\ \hat{RA}=RL\\ \hat{LA}=LL\\ \hat{C_{X}}=C_{X} \end{array}\right]$	$\left[\begin{array}{cccc} 0 & 0 & 1 & 0\\ 1 & 0 & 0 & 0\\ 1 & 0 & 0 & 0\\ 0 & 0 & 0 & 1 \end{array}\right]$	$\left[\begin{array}{ccc} 0 & 0 & 0\\ 1 & -1 & 0\\ 0 & -1/3 & 1 \end{array}\right]$	$\hat{I}=0$ $\hat{II}=I-II=-III$ ${\hat{V}}_{X}=V_{X}-1/3II$
Cross rotation $\hat{\mathrm{RL}}$→$\hat{\mathrm{RA}}$→$\hat{\mathrm{LL}}$→$\hat{\mathrm{LA}}$→$\hat{\mathrm{RL}}$			
$\left[\begin{array}{c} \hat{LL}=RA\\ \hat{RA}=RL\\ \hat{LA}=LL\\ \hat{C_{X}}=C_{X} \end{array}\right]$	$\left[\begin{array}{cccc} 0 & 1 & 0 & 0\\ 1 & 0 & 0 & 0\\ 1 & 0 & 0 & 0\\ 0 & 0 & 0 & 1 \end{array}\right]$	$\left[\begin{array}{ccc} 0 & 0 & 0\\ 0 & -1 & 0\\ 1/3 & -1/3 & 1 \end{array}\right]$	$\hat{I}=0$ $\hat{II}=-II$ ${\hat{V}}_{X}=V_{X}-1/3\left(II-I\right)$

*Note:* All columns correspond to the description in the footer of Table 2.

**Table 4:** Reversals of unicolor chest and peripheral electrodes.

Reversed Electrodes	S	$MS{\tilde{M}}_{inv}$	Reordered Leads
Red electrodes $\hat{\mathrm{RA}}$↔$\hat{C1}$			
$\left[\begin{array}{c} \hat{LL}=LL\\ \hat{RA}=C_{1}\\ \hat{LA}=LA\\ \hat{C_{1}}=RA\\ \hat{C_{X}}=C_{X} \end{array}\right]$	$\left[\begin{array}{ccccc} 1 & 0 & 0 & 0 & 0\\ 0 & 0 & 0 & 1 & 0\\ 0 & 0 & 1 & 0 & 0\\ 0 & 1 & 0 & 0 & 0\\ 0 & 0 & 0 & 0 & 1 \end{array}\right]$	$\left[\begin{array}{cccc} 2/3 & -1/3 & -1 & 0\\ -1/3 & 2/3 & -1 & 0\\ -4/9 & -4/9 & -1/3 & 0\\ -1/9 & -1/9 & -1/3 & 1 \end{array}\right]$	$\hat{I}=\left(I-III\right)/3-V_{1}$ $\hat{II}=\left(II+III\right)/3-V_{1}$ ${\hat{V}}_{1}=-\frac{4}{9}\left(I+II\right)-\frac{1}{3}V_{1}$ ${\hat{V}}_{X}=V_{X}-\frac{1}{9}\left(I+II\right)-\frac{1}{3}V_{1}$
Yellow electrodes $\hat{\mathrm{LA}}$↔$\hat{C2}$			
$\left[\begin{array}{c} \hat{LL}=LL\\ \hat{RA}=RA\\ \hat{LA}=C_{2}\\ \hat{C_{2}}=LA\\ \hat{C_{X}}=C_{X} \end{array}\right]$	$\left[\begin{array}{ccccc} 1 & 0 & 0 & 0 & 0\\ 0 & 1 & 0 & 0 & 0\\ 0 & 0 & 0 & 1 & 0\\ 0 & 0 & 1 & 0 & 0\\ 0 & 0 & 0 & 0 & 1 \end{array}\right]$	$\left[\begin{array}{cccc} 1/3 & 1/3 & 1 & 0\\ 0 & 1 & 0 & 0\\ 8/9 & -4/9 & -1/3 & 0\\ 2/9 & -1/9 & -1/3 & 1 \end{array}\right]$	$\hat{I}=\frac{1}{3}\left(I+II\right)+V_{2}$ $\hat{II}=II$ ${\hat{V}}_{2}=\frac{4}{9}\left(I-III\right)-\frac{1}{3}V_{2}$ ${\hat{V}}_{X}=V_{X}+\frac{1}{9}\left(I-III\right)-\frac{1}{3}V_{1}$
Green electrodes $\hat{\mathrm{LL}}$↔$\hat{C3}$			
$\left[\begin{array}{c} \hat{LL}=C_{3}\\ \hat{RA}=RA\\ \hat{LA}=LA\\ \hat{C_{3}}=LL\\ \hat{C_{X}}=C_{X} \end{array}\right]$	$\left[\begin{array}{ccccc} 0 & 0 & 0 & 1 & 0\\ 0 & 1 & 0 & 0 & 0\\ 0 & 0 & 1 & 0 & 0\\ 1 & 0 & 0 & 0 & 0\\ 0 & 0 & 0 & 0 & 1 \end{array}\right]$	$\left[\begin{array}{cccc} 1 & 0 & 0 & 0\\ 1/3 & 1/3 & 1 & 0\\ -4/9 & 8/9 & -1/3 & 0\\ -1/9 & 2/9 & -1/3 & 1 \end{array}\right]$	$\hat{I}=I$ $\hat{II}=\frac{1}{3}\left(I+II\right)+V_{3}$ ${\hat{V}}_{3}=\frac{4}{9}\left(II+III\right)-\frac{1}{3}V_{3}$ ${\hat{V}}_{X}=V_{X}+\frac{1}{9}\left(II+III\right)-\frac{1}{3}V_{1}$
Black electrodes $\hat{\mathrm{RL}}$↔$\hat{C5}$			
$\left[\begin{array}{c} \hat{LL}=LL\\ \hat{RA}=RA\\ \hat{LA}=LA\\ \hat{C_{5}}=RL\\ \hat{C_{X}}=C_{X} \end{array}\right]$	$\left[\begin{array}{ccccc} 1 & 0 & 0 & 0 & 0\\ 0 & 1 & 0 & 0 & 0\\ 0 & 0 & 1 & 0 & 0\\ 1 & 0 & 0 & 0 & 0\\ 0 & 0 & 0 & 0 & 1 \end{array}\right]$	$\left[\begin{array}{cccc} 1 & 0 & 0 & 0\\ 0 & 1 & 0 & 0\\ -1/3 & 2/3 & 0 & 0\\ 0 & 0 & 0 & 1 \end{array}\right]$	$\hat{I}=I$ $\hat{II}=II$ ${\hat{V}}_{5}=\left(II+III\right)/3$ ${\hat{V}}_{X}=V_{X}$
All unicolor electrode pairs $\hat{\mathrm{RA}}$↔$\hat{C1}$, $\hat{\mathrm{LA}}$↔$\hat{C2}$, $\hat{\mathrm{LL}}$↔$\hat{C3}$, $\hat{\mathrm{RL}}$↔$\hat{C5}$			
$\left[\begin{array}{c} \hat{LL}=C_{3}\\ \hat{RA}=C_{1}\\ \hat{LA}=C_{2}\\ \hat{C_{1}}=RA\\ \hat{C_{2}}=LA\\ \hat{C_{3}}=LL\\ \hat{C_{4}}=C_{4}\\ \hat{C_{5}}=RL\\ \hat{C_{6}}=C_{6} \end{array}\right]$	$\left[\begin{array}{ccccccccc} 0 & 0 & 0 & 0 & 0 & 1 & 0 & 0 & 0\\ 0 & 0 & 0 & 1 & 0 & 0 & 0 & 0 & 0\\ 0 & 0 & 0 & 0 & 1 & 0 & 0 & 0 & 0\\ 0 & 1 & 0 & 0 & 0 & 0 & 0 & 0 & 0\\ 0 & 0 & 1 & 0 & 0 & 0 & 0 & 0 & 0\\ 1 & 0 & 0 & 0 & 0 & 0 & 0 & 0 & 0\\ 0 & 0 & 0 & 0 & 0 & 0 & 1 & 0 & 0\\ 1 & 0 & 0 & 0 & 0 & 0 & 0 & 0 & 0\\ 0 & 0 & 0 & 0 & 0 & 0 & 0 & 0 & 1 \end{array}\right]$	$\left[\begin{array}{cccccccc} 0 & 0 & -1 & 1 & 0 & 0 & 0 & 0\\ 0 & 0 & -1 & 0 & 1 & 0 & 0 & 0\\ -\frac{1}{3} & -\frac{1}{3} & -\frac{1}{3} & -\frac{1}{3} & -\frac{1}{3} & 0 & 0 & 0\\ \frac{2}{3} & -\frac{1}{3} & -\frac{1}{3} & -\frac{1}{3} & -\frac{1}{3} & 0 & 0 & 0\\ -\frac{1}{3} & \frac{2}{3} & -\frac{1}{3} & -\frac{1}{3} & -\frac{1}{3} & 0 & 0 & 0\\ 0 & 0 & -\frac{1}{3} & -\frac{1}{3} & -\frac{1}{3} & 1 & 0 & 0\\ -\frac{1}{3} & \frac{2}{3} & -\frac{1}{3} & -\frac{1}{3} & -\frac{1}{3} & 0 & 0 & 0\\ 0 & 0 & -\frac{1}{3} & -\frac{1}{3} & -\frac{1}{3} & 0 & 0 & 1 \end{array}\right]$	$\hat{I}=V_{2}-V_{1}$ $\hat{II}=V_{3}-V_{1}$ ${\hat{V}}_{1}=-\frac{I+II}{3}-\frac{V_{1}+V_{2}+V_{3}}{3}$ ${\hat{V}}_{2}=\frac{I-III}{3}-\frac{V_{1}+V_{2}+V_{3}}{3}$ ${\hat{V}}_{3}=\frac{II+III}{3}-\frac{V_{1}+V_{2}+V_{3}}{3}$ ${\hat{V}}_{4}=V_{4}-\frac{V_{1}+V_{2}+V_{3}}{3}$ ${\hat{V}}_{5}=\frac{II+III}{3}-\frac{V_{1}+V_{2}+V_{3}}{3}$ ${\hat{V}}_{6}=V_{6}-\frac{V_{1}+V_{2}+V_{3}}{3}$

*Note:* All columns correspond to the description in the footer of Table 2. Cx represents all chest electrodes with unchanged positions, Vx denotes their unipolar leads.

**Table 5:** Statistical analysis (mean value ± standard deviation) of measures (RMS Error, Peak Error, CorCoef) calculated for the average beat in 8 independent leads (I, II, V1-V6), quantifying the lead-specific differences between experimentally recorded ECGs – raw leads vs. reordered leads (based on the expressions in Table 4) for 3 different scenarios:. No transformation; Forward ‘MSMinv’ transformation applied on the recording with correct electrodes; Inverse ‘MSMinv’ transformation applied on the recording with swapped electrodes.

Unicolor Reversals		RMS Error (µV)			Peak Error (µV)			Cor. Coef. (0–1)		
		Transf. None	Transf. MSMinv Forward	Transf. MSMinv Inverse	Transf. None	Transf. MSMinv Forward	Transf. MSMinv Inverse	Transf. None	Transf. MSMinv Forward	Transf. MSMinv Inverse
RA-C1	**I**	81 ± 40	14 ± 5 *	15 ± 5 *	351 ± 162	48 ± 19 *	53 ± 20 *	0.921 ± 0.093	0.997 ± 0.004 *	0.995 ± 0.006 *
	**II**	81 ± 38	13 ± 5 *	13 ± 4 *	356 ± 163	48 ± 19 *	46 ± 16 *	0.909 ± 0.172	0.998 ± 0.002 *	0.996 ± 0.005 *
	**V1**	120 ± 52	11 ± 4 *	11 ± 4 *	529 ± 227	38 ± 15 *	38 ± 13 *	0.832 ± 0.136	0.996 ± 0.002 *	0.998 ± 0.002 *
	**Vx**	27 ± 13	11 ± 5 *	12 ± 5 *	116 ± 61	44 ± 9 *	42 ± 18 *	0.989 ± 0.029	0.998 ± 0.003 *	0.998 ± 0.003 *
LA-C2	**I**	197 ± 80	16 ± 5 *	15 ± 5 *	924 ± 336	55 ± 20 *	54 ± 20 *	0.746 ± 0.144	0.997 ± 0.004 *	0.995 ± 0.005 *
	**II**	18 ± 5	13 ± 3 *	13 ± 3 *	78 ± 37	44 ± 12 *	46 ± 13 *	0.994 ± 0.013	0.995 ± 0.011 *	0.995 ± 0.009 *
	**V2**	246 ± 102	13 ± 6 *	13 ± 5 *	1121 ± 396	50 ± 25 *	54 ± 26 *	0.456 ± 0.180	0.990 ± 0.010 *	0.998 ± 0.002 *
	**Vx**	58 ± 5	12 ± 4 *	12 ± 5 *	264 ± 103	41 ± 16 *	47 ± 19 *	0.956 ± 0.073	0.998 ± 0.003 *	0.997 ± 0.003 *
LL-C3	**I**	15 ± 5	15 ± 5	15 ± 6	56 ± 27	54 ± 23	56 ± 26	0.996 ± 0.006	0.995 ± 0.006 *	0.995 ± 0.006 *
	**II**	154 ± 50	18 ± 5*	15 ± 4 *	644 ± 228	59 ± 22 *	53 ± 20 *	0.869 ± 0.146	0.998 ± 0.001 *	0.995 ± 0.008 *
	**V3**	206 ± 65	12 ± 5 *	14 ± 5 *	868 ± 296	47 ± 25 *	53 ± 21 *	0.512 ± 0.197	0.988 ± 0.012 *	0.997 ± 0.004 *
	**Vx**	52 ± 18	12 ± 5 *	13 ± 5 *	219 ± 80	45 ± 23 *	46 ± 20 *	0.970 ± 0.051	0.997 ± 0.004 *	0.997 ± 0.003*
RL-C5	**I**	16 ± 5	15 ± 5	15 ± 5	57 ± 24	54 ± 20	54 ± 20	0.995 ± 0.008	0.995 ± 0.008	0.995 ± 0.009
	**II**	14 ± 4	14 ± 4	14 ± 4	47 ± 15	48 ± 18	48 ± 17	0.995 ± 0.009	0.995 ± 0.009	0.995 ± 0.010
	**V5**	131 ± 60	24 ± 12 *$	136 ± 54 #	639 ± 287	102 ± 54 *$	660 ± 268 #	0.785 ± 0.210	0.957 ± 0.047 *$	0.792 ± 0.144 #
	**Vx**	14 ± 5	14 ± 5 *	14 ± 5 *	51 ± 25	51 ± 25	51 ± 25	0.998 ± 0.002	0.998 ± 0.002	0.997 ± 0.002
RA-C1 LA-C2 LL-C3 RL-C5	**I**	143 ± 72	17 ± 9 *	18 ± 8 *	726 ± 361	59 ± 33 *	69 ± 35 *	0.836 ± 0.100	0.994 ± 0.006 *	0.994 ± 0.005 *
	**II**	138 ± 45	19 ± 5 *	18 ± 9 *	631 ± 275	71 ± 26 *	60 ± 27 *	0.925 ± 0.062	0.997 ± 0.002 *	0.993 ± 0.009 *
	**V1**	225 ± 97	16 ± 5 *	14 ± 5 *	967 ± 445	50 ± 17 *	57 ± 18 *	0.652 ± 0.195	0.996 ± 0.003 *	0.997 ± 0.003 *
	**V2**	289 ± 125	16 ± 7 *	18 ± 8 *	1235 ± 487	62 ± 35 *	69 ± 30 *	0.365 ± 0.133	0.993 ± 0.007 *	0.996 ± 0.004 *
	**V3**	248 ± 92	15 ± 5 *	16 ± 4 *	1003 ± 378	55 ± 19 *	58 ± 23 *	0.494 ± 0.184	0.994 ± 0.010 *	0.997 ± 0.003*
	**V4**	110 ± 56	14 ± 4 *	16 ± 4 *	443 ± 258	54 ± 20 *	58 ± 25 *	0.835 ± 0.204	0.997 ± 0.003 *	0.997 ± 0.002 *
	**V5**	161 ± 63	26 ± 10 *$	142 ± 75 #	615 ± 285	104 ± 50*$	659 ± 368 #	0.705 ± 0.230	0.986 ± 0.019 *$	0.859 ± 0.106 #
	**V6**	113 ± 53	13 ± 5 *	13 ± 5 *	472 ± 231	50 ± 23 *	45 ± 16 *	0.886 ± 0.135	0.998 ± 0.002 *	0.996 ± 0.005 *

*Note: Vx* denotes the unipolar chest leads without electrode swaps; Color of electrodes follows IEC standard [44]; Gray columns highlight the baseline measurements without transformation (‘None’ transformation). * *p* < 0.05: Significant reduction of (RMS Error, Peak Error) and increment of (CorCoef) for the reordered leads after applying ‘MSMinv’ transformation (Forward or Inverse) compared to ‘None’ transformation. $: Approximation effect of the Forward ‘MSMinv’ transformation to simulate a swapped lead V5, while C5 is placed on the RL and its potential is assumed equal to the LL. #: Deficiency of the Inverse ‘MSMinv’ transformation to reconstruct the correct lead V5 from a recording with RL electrode in the position of C5 electrode.

**Table 6:** Accuracy for the detection of the exact lead swaps, applying the ‘MSMinv’ transformation on 3 swapped recordings (RA-C1), (LA-C2), (LL-C3) from 25 patients in our database. Color of electrodes follows IEC standard [44];

	Accuracy		
Unicolor Lead Swaps	RMS Error (%)	Peak Error (%)	Cor Coef (%)
RA-C1	100	96 *	100
LA-C2	96 *	88 #	96 *
LL-C3	100	88 #	100

* 1 false negative case due to TLS with the 2nd ranked minimal difference in the decision rule (17). # 3 false negative cases due to TLS with either the 2nd or the 3rd ranked minimal differences in (17).
